# Genetic Attributes and Conservation of an Endangered Giant Water Bug Species, *Diplonychus esakii* Miyamoto and Lee, 1966 (Hemiptera: Belostomatidae)

**DOI:** 10.3390/insects15100754

**Published:** 2024-09-29

**Authors:** Seon Yi Kim, Changseob Lim, Ji Hyoun Kang, Yeon Jae Bae

**Affiliations:** 1 Department of Life Sciences, Graduate School, Korea University, Seoul 02841, Republic of Korea; paimon@korea.kr; 2 Species Diversity Research Division, Biodiversity Research Department, National Institute of Biological Resources, Incheon 22689, Republic of Korea; 3 Korean Entomological Institute, Korea University, Seoul 02841, Republic of Korea; stagbeetle95@korea.ac.kr; 4 Division of Environmental Science and Ecological Engineering, College of Life Sciences and Biotechnology, Korea University, Seoul 02841, Republic of Korea

**Keywords:** conservation biology, genetic diversity, Korean Peninsula, mitochondrial DNA, population structure, top predator

## Abstract

**Simple Summary:**

An endangered giant water bug species, *Diplonychus esakii,* is one of the top predators in Korean freshwater ecosystems. This study investigates *D. esakii* populations in South Korea, a species potentially endemic to this region, and identifies 11 haplotypes with a haplotype diversity value of 0.623 out of 318 individuals across 27 sites. Through AMOVA (analysis of molecular variance) and *F*_ST_ analyses, we discovered significant genetic differentiation among populations and limited gene flow, indicating potential vulnerability to environmental changes. Consequently, we emphasize the need for conservation efforts to protect *D. esakii*. We also highlight the value of the Upo Wetland and Jeju Island populations as important conservation units to conserve the genetic diversity of Korean *D. esakii*. We also suggest an evaluation of the conservation status of *D. esakii* compared to the level of genetic diversity known from other endangered insect species. The genetic information in this study will provide valuable data for developing effective conservation strategies.

**Abstract:**

*Diplonychus esakii*, a water bug from the family Belostomatidae, plays an important role in freshwater ecosystems as one of the top predators. In this study, we investigated the genetic diversity and population structure of *D. esakii* by analyzing 318 specimens across 27 sites in South Korea. We found that the populations of *D. esakii* possess 11 haplotypes with a haplotype diversity of 0.623. This represents a relatively low level of genetic diversity compared to other known belostomatids and endangered species. AMOVA and *F*_ST_ analyses revealed significant genetic differentiation among populations, with most populations harboring only 1–2 haplotypes, suggesting restricted gene flow between populations and a low level of genetic diversity. This low genetic diversity and limited gene flow suggest a potential vulnerability to environmental changes and an increased risk of extinction, indicating that *D. esakii* should be designated as a protected species in South Korea as part of future conservation efforts. Based on the results of this study, Upo Wetland, which maintains relatively high levels of genetic diversity and Jeju Island, which, despite its lower genetic diversity compared to the mainland, does not share haplotypes with other regions, should be considered key conservation units for this species. This study highlights the importance of incorporating genetic information into conservation status assessments under the Red List Categories and Criteria and also emphasizes the need to evaluate this species on the Korean Red List. The data provided here will serve as essential baseline information and valuable resources for the development of effective conservation strategies.

## 1. Introduction

*Diplonychus esakii* Miyamoto and Lee, 1966, belongs to the family Belostomatidae (Heteroptera: Hemiptera) and is commonly known as a giant water bug. This species serves as a top predator in aquatic ecosystems [1,2,3], preying on a variety of aquatic organisms including insects, amphibians, and small fish [4,5,6,7,8,9,10]. Typically, they are observed at the water’s edge, positioning their forelegs downward and their abdominal spiracles above the water surface [5].

As top predators in wetland ecosystems, giant water bugs exhibit several traits that make them vulnerable to environmental changes, such as ecological dependence on prey species and relatively low fecundity [11]. *Diplonychus esakii*, a relatively large-sized predator with limited dispersal capability and confined to specific microhabitats in geographically isolated areas [1], potentially meets the criteria for ‘endangered status’ in South Korea. Moreover, aquatic insects like *D. esakii* face extinction risks due to threats to aquatic habitats, necessitating conservation efforts closely tied to wetland habitat protection. As of 2022, the total wetland area identified in South Korea amounted to 3635.6 km^2^, representing approximately 3.6% of the country’s total land area. According to the 4th National Inland Wetland Monitoring Report (2016–2020), 176 out of 2499 wetlands were confirmed to have been lost due to reclamation, cultivation, and development activities. The degradation of wetland habitats, exacerbated by urbanization, industrialization, and climate change, is contributing to a significant decline in biodiversity [12].

*Diplonychus esakii* is considered to have a more restricted distribution than previously reported, potentially being endemic to South Korea [13,14,15,16]. The type locality of *D. esakii* is Jeju Island [17]. The distribution of the genus *Diplonychus* is reported to cover Africa, southern Asia, the Orient, the East Indies, and Australia [18], while *D. esakii* has been recorded in South Korea, Japan, Taiwan, and the Oriental regions [19]. However, the Illustrated Encyclopedia of Fauna & Flora of Korea [13] only records its distribution in South Korea, and *D. esakii* is absent from Aquatic Insects of Japan, which instead lists *D. rusticus* [14]. No specimens or published data support its presence in Taiwan, and the species identification in Chinese sources remains uncertain [15]. Furthermore, sequences from Vietnamese specimens [16] obtained from NCBI GenBank suggest that they are more closely related to *D. rusticus* than to *D. esakii*. In South Korea, *D. esakii* predominantly inhabits southern regions, raising the possibility that this species has an extremely limited global distribution.

The Upo Wetland, recognized for its ecological importance and protected as the largest natural inland wetland in South Korea, formed from narrowed streams flowing into the Nakdong River, spanning approximately 2.5 km in width and 1.6 km in length. As an intermediate stage in ecological succession transitioning to terrestrial environments, Upo Wetland exhibits high biodiversity and ecological resilience [20]. From 2006 to 2013, a survey of benthic macroinvertebrates in Upo Wetland identified *D. esakii* as one of the most dominant insects [21]. Jeju Island, another region with a concentration of Ramsar wetlands, hosts 5 of South Korea’s 24 designated Ramsar sites [22]. These five wetlands, recognized for their ecological significance, support numerous endangered species and exhibit a high biodiversity [23]. A survey of aquatic insects in these Jeju wetlands revealed the presence of *D. esakii* in 32 out of 102 sites [24]. Despite ongoing surveys of aquatic insects for wetland conservation and the occurrence of *D. esakii* [25], the genetic attributes such as the genetic diversity and population genetic structure of *D. esakii* remain unknown.

Understanding the level of genetic diversity and population structure of *D. esakii* is essential for assessing its current conservation status, particularly in the face of habitat changes and environmental stressors [26]. The level of genetic diversity within a species is a critical factor that determines its ability to adapt to environmental changes, resist diseases, and maintain overall population viability [27]. A high level of genetic diversity typically indicates a robust population capable of withstanding various ecological pressures, whereas the low level of genetic diversity may suggest vulnerability to environmental fluctuations and a higher risk of extinction [28,29,30].

The haplotype diversity of the COI gene refers to the variability in the genetic sequences of a specific region of DNA, often used to infer the historical and evolutionary dynamics of populations. In population genetics, haplotype data provide insights into the genetic variation and connectivity among populations [26]. For aquatic insects like *D. esakii*, haplotype diversity can reveal patterns of dispersal, breeding behavior, and historical population sizes, which are essential for designing effective conservation strategies [31,32].

The study of population genetics involves examining the genetic composition of populations and how it changes over time due to factors like mutation, selection, genetic drift, and gene flow [29]. In the context of aquatic insects, population genetic studies help to elucidate the impacts of habitat fragmentation, pollution, and climate change on genetic connectivity. For *D. esakii*, understanding these genetic dynamics can aid in assessing the health of populations and the ecosystems they inhabit [26].

Within the same family of Belostomatidae, *Kirkaldyia deyrolli*, designated as an endangered species by the Ministry of Environment in 2007, is currently the focus of a restoration project in Chungcheong-do, with ongoing releases. Prior studies on the genetic diversity of *K. deyrolli* have been conducted [33,34,35,36], but this research marks the first study on the genetic diversity and population structure of *D. esakii* in South Korea.

Two species of Coleoptera, *Callipogon relictus* and *Copris tripartitus*, along with one species of Odonata, *Nannophya koreana*, are designated as endangered species by the Ministry of Environment and are categorized in the Korean Red List as critically endangered (CR), least concern (LC), and vulnerable (VU), respectively (Table 1). *Kirkaldyia deyrolli*, which has not been evaluated in the Korean Red List, and is classified as vulnerable (VU) in Japan, exhibits a lower genetic diversity compared to the other three species (Table 1). The data of the levels of genetic diversity of belostomatids from other regions (e.g., the Japanese population of *K. deyrolli,* and *A. japonicus*) (Table 1) can be a valuable reference for assessing both the Korean giant water bug *K. deyrolli* and *D. esakii*.

In this study, we aim to investigate the current conservation status of the Korean *D. esakii* by examining its level of genetic diversity and population structure, thereby contributing to the establishment of regional conservation units and the formulation of effective conservation strategies.

## 2. Materials and Methods

### 2.1. Sample Collection and COI Gene Sequencing

**Figure 1:**
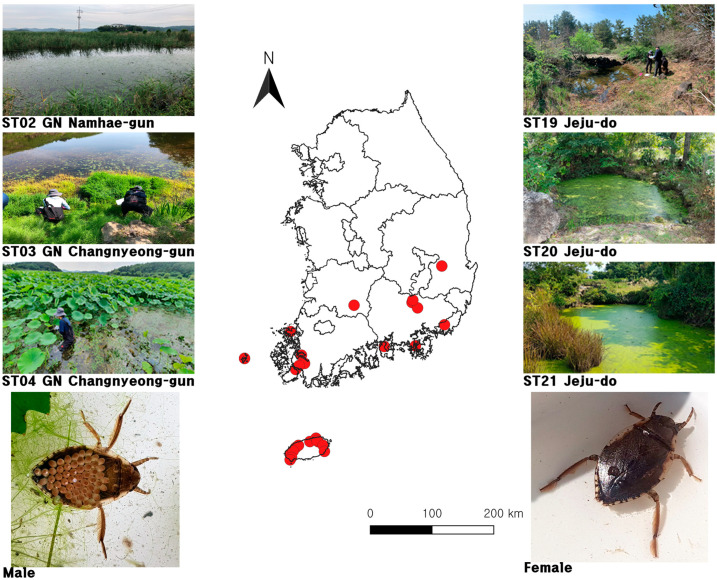
Map of 27 sampling sites of natural *Diplonychus esakii* populations from South Korea. The map was modified from a version produced using QGIS v.3.34.5 [46].

The present study included a total of 318 specimens that had been collected from 27 different sampling sites in South Korea (Figure 1). These specimens were fixed with 95% ethanol. Detailed information on all specimens is shown in Table 2.

Total genomic DNA was extracted from leg tissue using the DNeasy Blood and Tissue Kit (Qiagen, Hilden, Germany) according to the manufacturer’s instructions. Total DNA was used to amplify DNA fragments by the polymerase chain reaction (PCR) with the universal primer set LCO1490/HCO2198 [47]. The PCR protocol was as follows: 94 °C for 1 min; 35 cycles of 94 °C for 1 min, 50 °C for 1 min, and 72 °C for 1 min; 72 °C for 7 min. The PCR products were visualized on a 1.5% agarose gel using UV light, purified enzymatically using Exonuclease I and Shrimp Alkaline Phosphatase (New England BioLabs, Ipswich, MA, USA), and sequenced by Bioneer Corp. Sequencing (Daejeon, Korea) using an ABI PRISM 3130xl Genetic Analyzer (Applied Biosystems, Foster city, CA, USA). The National Center for Biotechnology Information (NCBI) and Biodiversity Information System [48] of National Institute of Biological Resources (NIBR, Incheon, Korea) provided the sequences of the cytochrome c oxidase subunit I (COI) genes of 16 individuals (MK926433, MN053257–MN053271) and 4 individuals (WBN0388169–WBN0388171, WBN0401997). Haplotype sequences of COI for *D. esakii* obtained in the current study were deposited in GenBank under accession numbers PP930937–PP930945.

### 2.2. Genetic Diversity Analyses and Haplotype Network

A total of 318 sequences of COI (643 bp) from *D. esakii* were aligned using Clustal W implemented in BioEdit v.7.0.1 [49] and manually edited in MEGA v.7.0 [50]. Genetic indices and Tajima’s *D* and Fu’s *Fs* statistics were estimated using ARLEQUIN v.3.5 [51].

The haplotypes of the 318 individuals of *D. esakii* were determined using NJ algorithms in DnaSP v.5 [52]. The genetic diversity indices including haplotype diversity (h) and nucleotide diversity (π) were estimated using ARLEQUIN v.3.5. A haplotype network was reconstructed using PopART v.1.7 [53].

### 2.3. A Hierarchial Analysis of Molecular Variance (AMOVA) Analysis

The spatial population genetic structure of *D. esakii* was assessed by a hierarchal analysis of molecular variance (AMOVA), implemented in ARLEQUIN v.3.5. Analyses were conducted exclusively on sites where more than five individuals were obtained. First, a total of 16 sites with 297 individuals were assigned to two geographical groups: Gyeongsangnam-do (GN) and Jeju Island (JJ). Second, the sites were categorized into three groups based on river systems: Seomjin River (SJ), Nakdong River (ND), and Jeju Island (JJ). The group composition, site information, number of individuals, and corresponding genetic indices are presented in Table 2 (Group). Total molecular variance was partitioned among groups (Fct = ‘inter-group’ genetic variation), populations within groups (Fsc = ‘intra-group’ genetic variation), and populations, regardless of groupings (Fst = ‘inter-population’).

## 3. Results

### 3.1. Genetic Diversity

A total of 11 haplotypes of COI genes of specimens from the 27 localities were obtained with a haplotype diversity (*h*) of 0.623. The number of haplotypes ranged from one to four, and the haplotype diversity ranged from 0.000 to 0.857 for each population.

Upo Wetland (ST05) exhibited the highest number of haplotypes (NH = 4) from seven individuals. Additionally, this site demonstrated the highest haplotype diversity, with a value of 0.857. Seogwipo-si in Jeju Island (ST27) showed the second highest haplotype diversity of 0.762, following ST05. The sites with three haplotypes, the same as ST27, were ST03 and ST24, with haplotype diversities of 0.511 and 0.368, respectively. ST03 is located in Changnyeong-gun, Gyeongsangnam-do, near Upo Wetland, which is in the same province as ST05. Although ST24 had a relatively high number of haplotypes in this study, its haplotype diversity was found to be comparatively low.

### 3.2. Haplotype Network Analysis

**Figure 2:**
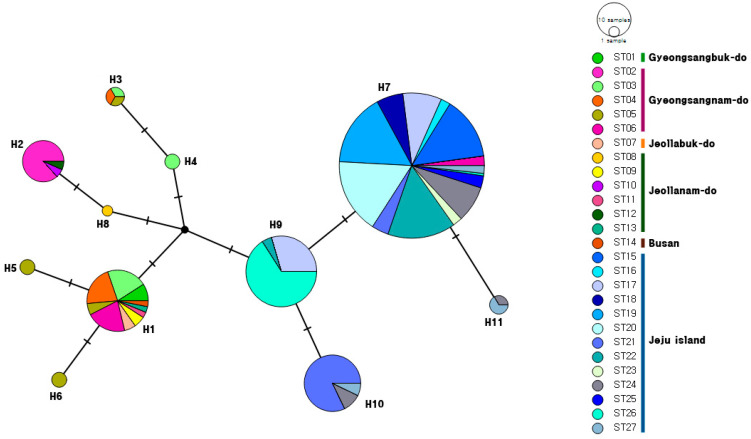
Haplotype network of COI sequences of *Diplonychus esakii* from 27 localities.

The most common haplotype (H7, *N* = 185) was shared among Jeju Island (ST15–ST27, *N* = 181) and Tongyeong-si, Gyeongsangnam-do (ST06, N = 4) (Figure 2).

The most abundant haplotype, H7, constitutes 58.2% of the total 318 individuals. The second most abundant haplotype, H9, comprises 44 individuals, accounting for 13.8%. These two haplotypes are predominantly found in the Jeju Island samples, excluding only four individuals (ST06). H1 (10.4%) is the third most abundant haplotype, encompassing the widest range of the regions (ST01: Yeongcheon, Gyeongsangbuk-do; ST03-ST05: Changnyeong, Gyeongsangnam-do; ST06: Tongyeong, Gyeongsangnam-do; ST07: Namwon, Jeollabuk-do; ST09: Sinan, Jeollanam-do; ST11: Jindo, Jeollanam-do; ST13: Haenam, Jeollanam-do; ST14: Sasang-gu, Busan).

Excluding regions with a sample size of five or fewer, we compared the haplotypes from five sites in Gyeongsangnam-do and eleven sites in Jeju Island. Among the total of eleven haplotypes, five sites in Gyeongsangnam-do (ST02–ST06) accounted for seven haplotypes (H1–H7), while eleven sites in Jeju Island (ST15, ST17–ST22, ST24–ST27) accounted for four haplotypes (H7, H9–H11). Jeju Island, except for H7, did not share haplotypes with the Gyeongsangnam-do region.

### 3.3. Population Genetic Differentiation

**Figure 3:**
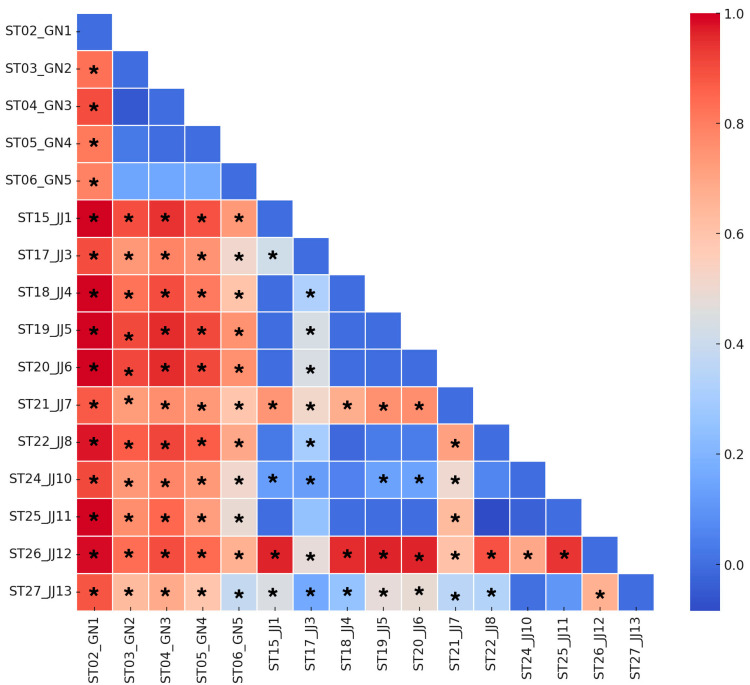
Pairwise *F*_ST_ values among the 16 populations of *Diplonychus esakii* (297 individuals). Significant *p*-values (*p* < 0.05) are indicated with asterisks (*).

The populations of *D. esakii* exhibited significant pairwise genetic differentiation (Figure 3), with *F*_ST_ values ranging from −0.084 to 1.000. Among these, considering only the significant values, the *F*_ST_ range was 0.125 to 1.000. The lowest value, 0.125, was observed between ST15 and ST24, both of which were populations within Jeju Island. ST02 exhibited significant differentiation from all 15 other sites, with *F*_ST_ values ranging from 0.790 to 1.000, indicating a high level of inter-population differentiation. The sites in Gyeongsangnam-do (ST03 to ST06) showed significant differentiation from all Jeju Island sites. Among the Jeju Island sites, ST21 and ST26 exhibited significant differences from all other sites, with *F*_ST_ values ranging from 0.353 to 0.873 and 0.476 to 0.985, respectively. Except for ST02, the populations in Gyeongsangnam-do (ST03 to ST06) generally did not exhibit significant differentiation from each other, whereas significant *F*_ST_ values, exceeding 0.500 overall, were observed between populations from Jeju Island and Gyeongsangnam-do. However, among the Gyeongsangnam-do populations, ST06 shared haplotypes with the Jeju Island populations ST25 and ST27, indicating values of 0.492 and 0.382, respectively, which were the lowest values observed between the Gyeongsangnam-do and Jeju Island populations.

### 3.4. Geographic Population Structure

The AMOVA analysis (Table 3) for Group I, which groups populations by two geographical regions, revealed significant genetic differentiation between them. These results indicate that the significant value of ΦCT and 64.04% of the total genetic variation can be attributed to the differences among regions, suggesting substantial genetic differentiation among two geographical regions. For Group II, 73.30% of the genetic variation is due to differences among the river systems, with a significant ΦCT value (0.733) reflecting strong genetic differentiation among river systems. Thus, the river system grouping analysis reveals strong genetic differentiations among river systems, indicating that riverine barriers may significantly influence genetic isolation.

## 4. Discussion

This study is the first to evaluate the genetic diversity of *D. esakii* across South Korea, which is likely the endemic region for the species.

Interestingly, *D. esakii* in South Korea exhibited a low level of genetic diversity and a distinct population structure with limited gene flow (Figure 3 and Table 3). The genetic diversity of *D. esakii* identified in this study is unexpectedly low compared to other belostomatid species, which are known to be at high conservation risk (Table 1) [35,37]. For example, *Kirkaldyia deyrolli*, an endangered species in South Korea, is now confined to only a few remaining habitats on Ganghwa Island and Jeju Island [54,55], with a haplotype diversity of 0.306 from 35 individuals, ranging from 0 to 0.833 among different populations. Another endangered beetle species, *Callipogon relictus*, along with a dung beetle species *Copris tripartitus* and a dragonfly species *Nannophya koreana*, exhibited haplotype diversity levels of 0.938, 0.962–1.000, and 0.946, respectively, demonstrating a higher genetic diversity than *D. esakii* despite their endangered status (Table 1).

Meanwhile, *A. japonicus* and *A. major*, which are protected species in Japan, exhibited haplotype diversities of 0.992 and 0.973, respectively, from samples collected in three countries, showing a higher level of genetic diversity compared to *D. esakii* [37]. However, a study that found a low haplotype diversity of 0.373 for *A. japonicus* analyzed a population isolated in the Matsumoto Basin of Nagano Prefecture, Japan [38]. *Diplonychus rusticus*, belonging to the same genus, exhibited a haplotype diversity of 0.922 across 54 individuals from seven populations across two countries [35], demonstrating a higher level of genetic diversity compared to *D. esakii*. In Thailand, *Lethocerus indicus* exhibited a haplotype diversity of 0.968 [35] and 0.932 [39], while *Belostoma angustum* in Brazil showed a haplotype diversity of 0.901 [40], indicating a higher level of genetic diversity than *D. esakii*.

The endangered and protected species mentioned above are managed under assessments from the Red List. Excluding *K. deyrolli*, which has not been evaluated, the Korean endangered species *C. relictus*, *C. tripartitus*, and *N. koreana* have been assessed as CR (critically endangered), LC (least concern), and VU (vulnerable), respectively, in the Korean Red List [56,57]. *A. japonicus*, which has exhibited low levels of haplotype diversity in Japanese populations [38], is also listed as NT (near threatened) on the Japanese Red List. Given the low genetic diversity of *D. esakii* compared to other protected species and the possibility that it may be confined to South Korea [13,14], contrary to its reported global distribution [19], it is important to conduct an objective evaluation of its conservation status to prioritize its protection.

There is a need to consider genetic information in the assessment of conservation status. The International Union for the Conservation of Nature (IUCN) Red List is a widely used tool for conservation assessment, utilizing information such as species’ range, population size, habitat quality and fragmentation, and abundance trends to assess extinction risk [58]. However, genetic diversity, which also affects extinction risk, is not currently considered [59]. According to the assessment guidelines currently used by the IUCN, the conservation status of *D. esakii* may not qualify for the threatened categories (i.e., protected species) because its geographic range—both the area of occupancy (AOO) and the extent of occurrence (EOO)—exceeds the thresholds and its insufficient evidence of habitat fragmentation, population decline, or range reduction for criteria B and D. Criteria A, C, and E require detailed data on population size and reduction (e.g., number of mature individuals, continuous decline over 10 years or 3 generations), which are often unavailable for most insect species, including *D. esakii*, making these criteria difficult to apply.

However, in terms of adaptive capacity, *D. esakii* may be more vulnerable than *K. deyrolli* or *N. koreana*, which are currently listed as endangered species in South Korea, despite having a wider distribution but lower genetic diversity (Table 2). When assessing vulnerability based on distribution data, particularly for insects where population decline is difficult to monitor, it is important to incorporate the genetic attributes of local populations with a distribution range to provide a more comprehensive understanding of species extinction risks [59].

The results of this study suggest that *D. esakii* populations are genetically distinct, with limited gene flow due to population isolation. The *F*_ST_ results indicate genetic differentiation among populations and regions. In particular, the genetic differentiation between Gyeongsangnam-do (GN) and Jeju Island (JJ) showed a more pronounced difference compared to that within Gyeongsangnam-do or within Jeju Island, demonstrating distinct geographic isolations between the regions. The significant differences observed in all sources of variation in the AMOVA, along with the highest percentage of variation occurring among groups, imply the presence of genetic differences between distinct groups. The low level of genetic diversity in this species has resulted in the presence of only 1–2 different haplotypes within each population, and the limited sharing of these haplotypes has led to the highest variation being observed among groups. Since each population possesses different genes and tends not to share unique haplotypes, leading to restricted gene flow, it is necessary to maintain and conserve the diversity of each *D. esakii* population. The loss of any population could result in the disappearance of its unique haplotypes, further reducing genetic diversity. Low genetic diversity indicates vulnerability to environmental changes [59,60] and can affect the risk of extinction [61]. Therefore, it is necessary to manage each population independently by designating them as separate conservation units [60].

Populations from two regions, Upo Wetland and Jeju Island, identified in our study need to be prioritized for protection from a conservation genetics perspective. According to the data on benthic macroinvertebrate communities, *D. esakii* was one of the most common species [21] and was reported as a dominant species in natural wetlands around Changnyeong-gun, including Upo Wetland [62]. Even if the level of genetic diversity across 318 individuals from 27 sites was relatively low, the ST05 population from Upo Wetland exhibited a slightly higher value (Table 2). Unlike other fragmented habitats of *D. esakii*, this species has established itself as a dominant species across a broader range within these wetlands [21,62]. The genetic analysis of populations from ST03 to ST05 in Changnyeong-gun, Gyeongsangnam-do, indicates a lack of significant genetic differentiation, suggesting potential gene flow among these populations. Given the geographical proximity and the possibility of movement between Upo Wetland (ST05) and nearby habitats (ST03–ST04), gene flow is likely. Upo Wetland is South Korea’s largest natural inland wetland, maintaining its primeval ecosystem while serving as a cradle of human life. It is a biodiversity hotspot, home to over 800 plant species, 209 bird species, 28 fish species, 180 macroinvertebrate species, and 17 mammal species [63]. Particularly, it provides a habitat for over 10 endangered species, including *Euryale ferox*, *Cygnus cygnus*, and *Mauremys reevesii*, underscoring its high biodiversity value [20]. Recognized for its ecological significance, Upo Wetland has been designated as a Ramsar wetland (Ramsar Convention, 1998) and a Wetland Protected Area (MOE, 1999). This study further emphasizes the value of Upo Wetland and proposes it as an appropriate population for the conservation of *D. esakii*, particularly for maintaining genetic diversity and facilitating gene flow.

The other important region is Jeju Island. Although only four haplotypes were identified in the Jeju Island region, none of those haplotypes are shared with the mainland of South Korea, except for four individuals at ST06, and the ST27 population from this region exhibited the second highest level of genetic diversity after Upo Wetland in our results (Table 2). Jeju Island has 11 crater lakes, including Baeknokdam in the Halla Mt., and over 150 small- to medium-sized inland wetlands [24,64]. Among the 24 Ramsar sites designated in South Korea, 5 are located on Jeju Island [22]. This island, where *D. esakii* is found not only in Ramsar wetlands but also in small ponds [24,65], is also the locality where the holotype of this species was collected [17]. Despite being the most densely populated region for *D. esakii* in South Korea, the populations on Jeju Island exhibit fewer haplotypes and a lower genetic diversity compared to those on the mainland, with a tendency not to share haplotypes with mainland populations. Moreover, significant genetic differentiation is observed among populations within Jeju Island. These population characteristics are likely to make the species vulnerable to environmental changes and extinction risk in this region [28,29,30]. Therefore, we propose designating Jeju Island as a distinct conservation unit for the protection of genetic diversity.

Mitochondrial DNA markers alone have successfully revealed the genetic diversity and structure in most studies on giant water bugs [37,38,39]. In particular, COI sequences have been considered suitable for investigating intraspecific genetic variation due to the presence of highly variable nucleotide sites in *L. indicus* in Thailand [39]. However, it should be noted that if two sexes differ in dispersal behavior, and only using mitochondrial genes can skew population structure analysis by reflecting female-only dispersal patterns. A study on the dispersal of *A. japonicus* suggested that during the breeding season, females may be more active dispersers than males, particularly those carrying eggs on their backs, even if the small sample size limited the ability to draw definitive conclusions [66]. However, clear sex-biased dispersal ability in *D. esakii* has not been identified thus far, and other belostomatid species are known to be capable of dispersal without significant differences between the sexes in flying and walking, particularly during dispersal events such as breeding seasons, floods, and pre-wintering periods [67,68,69,70]. Therefore, mtDNA may adequately reflect the genetic diversity of *D. esakii* in this study. Nevertheless, understanding genetic structure in the context of sex-specific dispersal behavior would benefit from future studies incorporating nuclear loci.

This study represents the first comprehensive analysis of genetic diversity and population structure of *D. esakii* in South Korea, encompassing the largest number of sampling sites and individuals. The findings reaffirm the high conservation values of populations from Upo Wetland and Jeju Island, such as their unique haplotypes and distinct population structures. By comparing the genetic diversity of *D. esakii* with that of other belostomatid species categorized as endangered in South Korea and other Asian regions, we have demonstrated that *D. esakii* has a relatively low genetic diversity with unique haplotypes for each population, suggesting that its conservation status may need to be evaluated for the Korean Red List. This study provides essential baseline data for the development of effective conservation strategies.

## Figures and Tables

**Table 1:** Levels of genetic diversity of insect species in critical conservation status from various studies.

Order	Family	Species	Region	Conservation Status	Conservation Status Record	*N*	NP	NH	*h* (total)	*h* (Pop.)	π (total)	π (pop.)	Marker	Ref.
Hemiptera	Belostomatidae	*Kirkaldyia deyrolli*	Korea/Japan	NE/VU Category II Endangered Species	Korean Red List (NE) Japanese Red List (VU)	35	5	5	0.306	0.000–0.833	0.001	0.000–0.071	COI	[35]
		*Appasus* *japonicus*	Japan/Korea/China	NT	Japanese Red List	143	67	103	0.992	0.500–1.000	0.021	0.000–0.014	COI + 16S	[37]
		*Appasus* *japonicus*	Japan	NT	Japanese Red List	530	33	26	0.373	0.100–0.667	0.001	0.000–0.018	COI	[38]
		*Appasus* *major*	Japan/Korea/Russia	NE	Japanese Red List	96	51	60	0.973	0.500–1.000	0.012	0.000–0.024	COI + 16S	[37]
		*Diplonychus rusticus*	Thailand /Philippines	-	-	54	7	38	0.922	0.750–1.000	0.011	0.006–0.017	COI	[35]
		*Lethocerus indicus*	Thailand	-	-	120	13	70	0.968	0.933–1.000	0.006	0.004–0.008	COI	[35]
			Thailand	-	-	90	12	37	0.932	0.700–1.000	0.005	0.002–0.007	COI	[39]
		*Belostoma angustum*	Brazil	-	-	98	18	42	0.901	0.600–1.000	0.003	0.002–0.005	COI	[40]
Coleoptera	Cerambycidae	*Callipogon relictus*	Korea /China /Russia	CR/ Category I Endangered Species	Korean Red List	32	12	19	0.938	0.000–1.000	-	0.000–0.025	COI	[41]
	Scarabaeidae	*Copris* *tripartitus*	Korea	LC/ Category II Endangered Species	Korean Red List	69	5	57	-	0.962–1.000	-	0.014–0.022	COI	[42]
Odonata	Libellulidae	*Nannophya koreana*	Korea	VU/ Category II Endangered Species	Korean Red List	11	4	9	0.946	1.000	0.003	0.000–0.005	COI	[43]
			Korea	VU/ Category II Endangered Species	Korean Red List	68	6	10	-	0.000–0.844	-	0.000–0.003	COI	[44]
			Korea	VU/ Category II Endangered Species	Korean Red List	108	5	14	-	0.000–0.874	-	0.000–0.003	COI	[45]

*N*: number of specimens, NP: number of populations, NH: number of haplotypes, *h*: haplotype diversity, π: nucleotide diversity, pop.: population. CR: critically endangered, VU: vulnerable, LC: least concern, NT: near threatened, NE: not evaluated.

**Table 2:** Sampling information and genetic indices of the *Diplonychus esakii* populations. Significant values (*p* < 0.05) are indicated in bold. Group abbreviations are as follows: Gyeongsangnam-do (GN), Seomjin River (SJ), Nakdong River (ND), and Jeju Island (JJ).

Site	Abbreviation	Group	Locality	GPS	*N*	NH	*h* (SD)	π (SD)	Tajima’s *D*	Fu’s *Fs*
ST01	GB		Yeongcheon-si, Gyeongsangbuk-do	36°02′ N/128°56′ E	3	1	0.000 (0.000)	0.000 (0.000)	0	-
ST02	GN1	GN SJ	Namhae-gun, Gyeongsangnam-do	34°52′ N/127°54′ E	13	1	0.000 (0.000)	0.000 (0.000)	0	-
ST03	GN2	GN ND	Changnyeong-gun, Gyeongsangnam-do	35°31′ N/128°23′ E	10	3	0.511 (0.164)	0.113 (0.091)	0.247	0.723
ST04	GN3	GN ND	Changnyeong-gun, Gyeongsangnam-do	35°26′ N/128°29′ E	8	2	0.250 (0.180)	0.075 (0.070)	−1.448	1.415
ST05	GN4	GN ND	Changnyeong-gun, Gyeongsangnam-do	35°33′ N/128°24′ E	7	4	0.857 (0.102)	0.181 (0.135)	−0.561	−0.324
ST06	GN5	GN ND	Tongyeong-si, Gyeongsangnam-do	34°53′ N/128°27′ E	11	2	0.509 (0.101)	0.153 (0.112)	1.681	3.360
ST07	JB		Namwon-si, Jeollabuk-do	35°28′ N/127°21′ E	2	1	0.000 (0.000)	0.000 (0.000)	0	-
ST08	JN1		Sinan-gun, Jeollanam-do	35°05′ N/126°14′ E	1	1	1.000 (0.000)	0.000 (0.000)	0	-
ST09	JN2		Sinan-gun, Jeollanam-do	34°41′ N/125°25′ E	2	1	0.000 (0.000)	0.000 (0.000)	0	-
ST10	JN3		Yeongam-gun, Jeollanam-do	34°44′ N/126°25′ E	1	1	1.000 (0.000)	0.000 (0.000)	0	-
ST11	JN4		Jindo-gun, Jeollanam-do	34°32′ N/126°19′ E	1	1	1.000 (0.000)	0.000 (0.000)	0	-
ST12	JN5		Haenam-gun, Jeollanam-do	34°37′ N/126°29′ E	1	1	1.000 (0.000)	0.000 (0.000)	0	-
ST13	JN6		Haenam-gun, Jeollanam-do	34°35′ N/126°23′ E	1	1	1.000 (0.000)	0.000 (0.000)	0	-
ST14	BS		Sasang-gu, Busan	35°11′ N/128°58′ E	1	1	1.000 (0.000)	0.000 (0.000)	0	-
ST15	JJ1	JJ	Jeju-si, Jeju-do	33°29′ N/126°44′ E	26	1	0.000 (0.000)	0.000 (0.000)	0	-
ST16	JJ2		Jeju-si, Jeju-do	33°29′ N/126°35′ E	4	1	0.000 (0.000)	0.000 (0.000)	0	-
ST17	JJ3	JJ	Jeju-si, Jeju-do	33°24′ N/126°21′ E	29	2	0.512 (0.031)	0.051 (0.050)	1.595	1.668
ST18	JJ4	JJ	Jeju-si, Jeju-do	33°26′ N/126°23′ E	11	1	0.000 (0.000)	0.000 (0.000)	0	-
ST19	JJ5	JJ	Jeju-si, Jeju-do	33°32′ N/126°42′ E	30	1	0.000 (0.000)	0.000 (0.000)	0	-
ST20	JJ6	JJ	Jeju-si, Jeju-do	33°30′ N/126°43′ E	31	1	0.000 (0.000)	0.000 (0.000)	0	-
ST21	JJ7	JJ	Jeju-si, Jeju-do	33°18′ N/126°16′ E	30	2	0.370 (0.084)	0.074 (0.063)	0.955	2.524
ST22	JJ8	JJ	Jeju-si, Jeju-do	33°18′ N/126°15′ E	30	2	0.129 (0.079)	0.013 (0.022)	−0.764	−0.439
ST23	JJ9		Jeju-si, Jeju-do	33°21′ N/126°18′ E	4	1	0.000 (0.000)	0.000 (0.000)	0	-
ST24	JJ10	JJ	Seogwipo-si, Jeju-do	33°15′ N/126°16′ E	19	3	0.368 (0.125)	0.067 (0.060)	−0.607	0.304
ST25	JJ11	JJ	Seogwipo-si, Jeju-do	33°13′ N/126°15′ E	5	1	0.000 (0.000)	0.000 (0.000)	0	-
ST26	JJ12	JJ	Seogwipo-si, Jeju-do	33°26′ N/126°49′ E	30	2	0.067 (0.061)	0.007 (0.016)	−1.147	−1.211
ST27	JJ13	JJ	Seogwipo-si, Jeju-do	33°21′ N/126°51′ E	7	3	0.762 (0.115)	0.143 (0.113)	0.755	0.668
	27 sites	16 sites			318	11	0.623 (0.027)	0.298 (0.190)	−0.539	−9.811

*N*: number of specimens, NH: number of haplotypes, *h*: haplotype diversity, π: nucleotide diversity.

**Table 3:** Analysis of molecular variance (AMOVA) of *Diplonychus esakii* in South Korea. Significant values (*p* < 0.01) are indicated in bold.

Grouping		Source of Variation	d.f.	Sum of Squares	Variance Components	Percentage of Variation	Φ-Statistics	*p*-Value
I	2 groups (by region)	Among groups	1	71.403	0.82598	64.04	ΦCT = **0.640**	<0.001
		Among populations within groups	14	75.207	0.27934	21.66	ΦSC = **0.602**	<0.001
		Within populations	281	51.815	0.18439	14.30	ΦST = **0.857**	<0.001
II	3 groups (by 3 river systems)	Among groups	2	94.831	1.05296	73.30	ΦCT = **0.733**	<0.001
		Among populations within groups	13	51.779	0.19914	13.86	ΦSC = **0.519**	<0.001
		Within populations	281	51.815	0.18439	12.84	ΦST = **0.872**	<0.001

## Data Availability

Data are available upon request from the authors.

## References

[1] Lytle D.A., Thorp J.H., Rogers D.C. (2015). Chapter 37 Order Hemiptera. Thorp and Covich’s Freshwater Invertebrates: Ecology and General Biology.

[2] Runck C., Blinn D.W. (1990). Population dynamics and secondary production by *Ranatra montezuma* (Heteroptera: Nepidae). J. N. Am. Benthol. Soc..

[3] Waters T.F. (1977). Secondary production in inland waters. Adv. Ecol. Res..

[4] Cullen M.J. (1969). The biology of giant water bugs (Hemiptera: Belostomatidae) in Trinidad. Proc. R. Entomol. Soc. Lond. A.

[5] Menke A.S. (1979). Family Belostomatidae: Giant water bugs, electric light bugs, toe biters. In: Menke AS (ed) The semiaquatic and aquatic Hemiptera of California (Heteroptera: Hemiptera). Bull. Calif. Insect Surv..

[6] Menke A.S. (1979). Family Nepidae: Water scorpions. In: Menke AS (ed) The semiaquatic and aquatic Hemiptera of California (Heteroptera: Hemiptera). Bull. Calif. Insect Surv..

[7] Okada H., Nakasuji F. (1993). Comparative studies on the seasonal occurrence, nymphal development and food menu in two giant water bugs, *Diplonychus japonicus* Vuillefroy and *Diplonychus major* Esaki (Hemiptera: Belostomatidae). Res. Popul. Ecol..

[8] Rankin K.P. (1935). Life history of *Lethocerus americanus* (Leidy) (Heteroptera: Belostomatidae). Univ. Kans. Sci. Bull..

[9] Smith R.L., Choe J.C., Crespi B.J. (1997). Evolution of parental care in the giant water bugs (Heteroptera: Belostomatidae). The Evolution of Social Behavior in Insects and Arachnids. II.

[10] Toledo L.F. (2003). Predation on seven South American anuran species by water bug (Belostomatidae). Phyllomedusa.

[11] Boersma K.S., Bogan M.T., Henrichs B.A., Lytle D.A. (2014). Top predator removals have consistent effects on large species despite high environmental variability. Oikos.

[12] Ministry of Environment (2022). The 4th National Wetland Conservation Plan 2023-2027.

[13] Lee C. (1971). True Bugs of Korea; Vol. 12, Animal Series (Insecta IV), Illustrated Encyclopedia of Fauna & Flora of Korea.

[14] Kawai T., Tanida K. (2018). Aquatic Insects of Japan: Manual with Keys and Illustrations.

[15] Ouyang X., Gao J., Chen B., Wang B., Ji H., Plath M. (2017). Charact. A Nov. Predat.–Prey Relatsh. Between Nativ. Diplonychus Esakii (Heteroptera: Belostomatidae) Invasive *Gambusia affinis* (Teleostei: Poeciliidae) Central China. Int. Aquat. Res..

[16] Ribeiro J.R.I., Ohba S.Y., Pluot-Sigwalt D., Stefanello F., Bu W., Meyin-A-Ebong S.E., Guilbert E. (2018). Phylogenetic Analysis and Revision of Subfamily Classification of Belostomatidae Genera (Insecta: Heteroptera: Nepomorpha). Zool. J. Linn. Soc..

[17] Miyamoto S., Lee C.E. (1966). Heteroptera of Quelpart Island (Chejudo). Sieboldia.

[18] Lauck D.R., Menke A.S. (1961). The Higher Classification of the Belostomatidae (Hemiptera). Ann. Entomol. Soc. Am..

[19] Polhemus J.T., Aukema B., Rieger C. (1995). Family Nepidae. Catalogue of the Heteroptera of the Palaearctic Region.

[20] Lee S.D. (2003). The Study of Current Status of Conservation and Management Policy on Wetlands in Korea. J. Wetl. Res..

[21] Kim H.G., Lee D.J., Yoon C.S., Cheong S.W. (2016). Assessing Biodiversity of Benthic Macroinvertebrates and Influences of Several Environmental Factors on the Community Structure in Upo Wetland by Long-term Ecological Monitoring. J. Environ. Sci. Int..

[22] National Institute of Ecology (NIE). https://www.nie.re.kr/nie/main/contents.do?menuNo=200291.

[23] Oh H.S., Yang H.G., Moon M., Park S.M., Banjad M., Cho M.H., Kim S.H., Kim H.K. (2023). The 1st Comprehensive Conservation Plan for Jeju Wetland Protection Area (2023–2027).

[24] Jeong S.B., Oh H.S., Jeon H.S., Yang K.S., Kim W.T. (2010). Aquatic Insects Fauna and Characteristics of Distribution on Jeju Island Wetlands. J. Wet. Res..

[25] National Institute of Ecology (NIE) (2023). The 5th Investigation of Natural Environment for Macroinvertebrates (2019–2022).

[26] Watanabe K., Koji S., Hidaka K., Nakamura K. (2013). Abundance, Diversity, and Seasonal Population Dynamics of Aquatic Coleoptera and Heteroptera in Rice Fields: Effects of Direct Seeding Management. Environ. Entomol..

[27] Gibson A.K. (2022). Genetic diversity and disease: The past, present, and future of an old idea. Evolution.

[28] Birader K. (2023). Genetic Diversity and the Adaptation of Species to Changing Environments. J Biodiv. Endan. Sp..

[29] Havemann N., Gossner M.M., Hendrich L., Morinière J., Niedringhaus R., Schäfer P., Raupach M.J. (2018). From water striders to water bugs: The molecular diversity of aquatic Heteroptera (Gerromorpha, Nepomorpha) of Germany based on DNA barcodes. PeerJ.

[30] Massa A.N., Sobolev V.S., Faustinelli P.C., Tallury S.P., Stalker H.T., Lamb M.C., Arias R.S. (2024). Genetic diversity, disease resistance, and environmental adaptation of *Arachis duranensis* L.: New insights from landscape genomics. PLoS ONE.

[31] Dong Z., Wang Y., Li C., Li L., Men X. (2021). Mitochondrial DNA as a Molecular Marker in Insect Ecology: Current Status and Future Prospects. Ann. Entomol. Soc. Amer..

[32] Pfeiler E., Markow T.A. (2017). Population connectivity and genetic diversity in long-distance migrating insects: Divergent patterns in representative butterflies and dragonflies. Bio. J. Linn Soc..

[33] Nakasako J., Okuyama H., Ohba S., Takahashi J. (2020). Complete mitochondrial DNA sequence of the giant water bug *Kirkaldyia deyrolli* (Hemiptera: Belostomatidae). Mito. DNA Part B.

[34] Sareein N., Kang J.H., Jung S.W., Phalaraksh C., Bae Y.J. (2019). Taxonomic review and distribution of giant water bugs (Hemiptera: Belostomatidae: Lethocerinae) in the Palearctic, Oriental, and Australian regions. Entomol. Res..

[35] Sareein N. (2019). Genetic diversity and conservation of Asian giant water bugs (Hemiptera: Belostomatidae). Ph.D. Thesis.

[36] Suzuki T., Ichiyanagi H., Ohba S. (2023). Discovery of a new population of the endangered giant water bug *Kirkaldyia deyrolli* (Heteroptera: Belostomatidae) in Kyushu and evaluation of their genetic structure. Entomol. Sci..

[37] Suzuki T., Kitano T., Tojo K. (2014). Contrasting genetic structure of closely related giant water bugs: Phylogeography of *Appasus japonicus* and *Appasus major* (Insecta: Heteroptera, Belostomatidae). Mol. Phylogenet. Evol..

[38] Tomita K., Suzuki T., Yano K., Tojo K. (2020). Community Structure of Aquatic Insects Adapted to Lentic Water Environments, and Fine-Scale Analyses of Local Population Structures and the Genetic Structures of an Endangered Giant Water Bug *Appasus japonicus*. Insects.

[39] Pradit N., Sumrandee C., Charernsom K., Chaiphongpachara T., Ruangchuay R., Ngernsoungnern A., Ngernsoungnern P. (2022). Genetic variation of the giant water bug *Lethocerus indicus* (Lepeletier and Serville, 1825) (Hemiptera: Belostomatidae) collected from natural habitats in northeastern Thailand. Aquat. Insects.

[40] Stefanello F., Menezes R.S.T., Ribeiro J.R.I., Almeida E.A.B. (2020). Widespread Gene Flow Model Explains the Genetic–Morphological Variation in a Giant Water Bug Species Under Fine-Scale Spatial Sampling. Ann. Entomol. Soc. Am..

[41] Kang J.H., Yi D.A., Kuprin A.V., Han C., Bae Y.J. (2021). Phylogeographic Investigation of an Endangered Longhorn Beetle, *Callipogon relictus* (Coleoptera: Cerambycidae), in Northeast Asia: Implications for Future Restoration in Korea. Insects.

[42] Kang A.R., Kim K.G., Park J.W., Kim I. (2012). Genetic diversity of the dung beetle, *Copris tripartitus* (Coleoptera: Scarabaeidae), that is endangered in Korea. Entomol. Res..

[43] Bae Y.J., Yum J.H., Kim D.G., Suh K.I., Kang J.H. (2020). *Nannophya koreana* sp. nov. (Odonata: Libellulidae): A new dragonfly species previously recognized in Korea as the endangered pygmy dragonfly *Nannophya pygmaea* Rambur. J. Spec. Res..

[44] Kim K.G., Jang S.K., Park D.W., Hong M.Y., Oh K.H., Kim K.Y., Hwang J.S., Han Y.S., Kim I.S. (2007). Mitochondrial DNA sequence variation of the tiny dragonfly, *Nannophya pygmaea* (Odonata: Libellulidae). Int. J. Indust. Entomol..

[45] Wang A.R., Kim M.J., Kim S.S., Kim I. (2017). Additional mitochondrial DNA sequences from the dragonfly, *Nannophya pygmaea* (Odonata: Libellulidae), which is endangered in South Korea. Int. J. Ind. Entomol..

[46] QGis QGis 3345 Zanzibar—AFree Open, G.I.S. https://qgis.org/tr/site/forusers/download.html.

[47] Folmer O., Black M., Hoeh W., Lutz R., Vrijenhoek R. (1994). DNA primers for amplification of mitochondrial cytochrome c oxidase subunit I from diverse metazoan invertebrates. Mol. Mar. Biol. Biotechnol..

[48] Biodiversity Information System. https://species.nibr.go.kr/ris/index.do.

[49] Hall T.A. (1999). BioEdit: A user-friendly biological sequence alignment editor and analysis program for Windows 95/98/NT. Nucleic Acids Symp. Ser..

[50] Kumar S., Stecher G., Tamura K. (2016). MEGA7: Molecular evolutionary genetics analysis version 7.0 for bigger datasets. Mol. Biol. Evol..

[51] Excoffier L., Laval G., Schneider S. (2005). Arlequin (version 3.0): An integrated software package for population genetics data analysis. Evol. Bioinform..

[52] Librado P., Rozas J. (2009). DnaSP v5: A software for comprehensive analysis of DNA polymorphism data. Bioinformatics.

[53] Bandelt H., Forster P., Röhl A. (1999). Median-joining networks for inferring intraspecific phylogenies. Mol. Bio. Evol..

[54] NIBR (2018). Biodiversity of the Five West Sea Islands.

[55] Joeng S.H. (2019). The Insects of Jeju Island Volume I.

[56] NIBR (2023). Red Data Book of Republic of Korea Insects II.

[57] NIBR (2023). Red Data Book of Republic of Korea Insects III.

[58] IUCN (2012). IUCN Red List Categories and Criteria, Version 3.1.

[59] Lim C., Kang J.H., Bae Y.J. (2024). Climate change will lead to range shifts and genetic diversity losses of dung beetles in the Gobi Desert and Mongolian Steppe. Sci. Rep..

[60] Matern A., Desender K., Drees C., Gaublomme E., Paill W., Assmann T. (2009). Genetic diversity and population structure of the endangered insect species *Carabus variolosus* in its western distribution range: Implications for conservation. Conserv. Genet..

[61] Schmidt C., Hoban S., Hunter M., Paz-Vinas I., Garroway C.J. (2023). Genetic diversity and IUCN Red List status. Conserv Biol..

[62] Park H.C., Kim W., Ri C.U. (1985). Flood and Adaptation of Insects at the Freshwater Wetland. Korean J. Ecol..

[63] Changnyeong-gun Upo Wetland. https://www.cng.go.kr/01656/01665/01715.web.

[64] Koh K.T. (2001). Wetlands of Jeju.

[65] Jeong S.W., Park Y.J., Ham S.A., Kim M.C., Oh H.S., Bae Y.J. (2011). Diversity and Species Composition of Benthic Macroinvertebrates in Jeju Island. Korean J. Entomol..

[66] Ohba S., Kato K., Miyatake T. (2010). Breeding ecology and seasonal abundance of the giant water bug *Appasus japonicus* (Heteroptera, Belostomatidae). Entomol. Sci..

[67] Lytle D.A. (1999). Use of rainfall cues by *Abedus herberti* (Hemiptera: Belostomatidae): A mechanism for avoiding flash floods. J. Insect Behav..

[68] Saijo H. (2001). Seasonal prevalence and migration of aquatic insects in paddies and an irrigation pond in Shimane Prefecture. Jpn. J. Ecol..

[69] Mukai Y., Baba N., Ishii M. (2005). The water system of traditional rice paddies as an important habitat of the giant water bug, *Lethocerus deyrollei* (Heteroptera: Belostomatidae). J. Ins. Conser..

[70] Mukai Y., Ishii M. (2007). Habitat utilization by the giant water bug, *Appasus* (=*Diplonychus*) *major* (Hemiptera: Belostomatidae), in a traditional rice paddy water system in northern Osaka, central Japan. Appl. Entomol. Zool..

